# Biliverdin/Bilirubin Redox Pair Protects Lens Epithelial Cells against Oxidative Stress in Age-Related Cataract by Regulating NF-*κ*B/iNOS and Nrf2/HO-1 Pathways

**DOI:** 10.1155/2022/7299182

**Published:** 2022-04-15

**Authors:** Yang Huang, Jinglan Li, Wenzhe Li, Nanping Ai, Haiying Jin

**Affiliations:** ^1^Department of Ophthalmology, Shanghai East Hospital, Tongji University School of Medicine, Shanghai, China; ^2^Senior Department of Ophthalmology, The Third Medical Center of PLA General Hospital, Beijing, China; ^3^School of Ophthalmology & Optometry, Wenzhou Medical University, Wenzhou, Zhejiang Province, China; ^4^Department of Basic Medicine, Changzhi Medical College, Changzhi, Shanxi Province, China

## Abstract

Age-related cataract (ARC) is the leading cause of vision impairment globally. It has been widely accepted that excessive reactive oxygen species (ROS) accumulation in lens epithelial cells (LECs) is a critical risk factor for ARC formation. Biliverdin (BV)/bilirubin (BR) redox pair is the active by-product of heme degradation with robust antioxidative stress and antiapoptotic effects. Thus, we purpose that BV and BR may have a therapeutic effect on ARC. In the present study, we determine the expression levels of enzymes regulating BV and BR generation in human lens anterior capsule samples. The therapeutic effect of BV/BR redox pair on ARC was assessed in hydrogen peroxide (H_2_O_2_)-damaged mouse LECs in vitro. The NF-*κ*B/inducible nitric oxide synthase (iNOS) and nuclear factor erythroid 2-related factor 2 (Nrf2)/heme oxygenase-1 (HO-1) signaling pathways were evaluated to illustrate the molecular mechanism. The results revealed that the mRNA expressions of Nrf2, HO-1, and biliverdin reductase A (BVRA) were all decreased in human samples of age-related nuclear cataract. BV/BR redox pair pretreatment protected LECs against H_2_O_2_ damage by prohibiting NF-*κ*B p65 nuclear trafficking, ameliorating iNOS expression, reducing intracellular and mitochondrial ROS levels, and restoring glutathione (GSH) and superoxide dismutase (SOD) levels. BV and BR pretreatment also regulated the expression of apoptotic molecules (Bax, Bcl-2, and cleaved caspase-3), thus decreasing the apoptosis of LECs. In addition, BV/BR pair promoted Nrf2 nuclear accumulation and HO-1 induction, whereas the knockdown of BVRA counteracted the effect of BV on activating Nrf2/HO-1 pathway and antiapoptosis. These findings implicated that BV/BR redox pair protects LECs against H_2_O_2_-induced apoptosis by regulating NF-*κ*B/iNOS and Nrf2/HO-1 pathways. Moreover, BVRA is responsible for BV-mediated cytoprotection by reductive conversion of BV to BR. This trial is registered with ChiCTR2000036059

## 1. Introduction

Age-related cataract (ARC) is the leading cause of reversible vision impairment globally with rising life expectancy and sociodemographic status and carries dramatic individual and societal burden [1]. ARC can be divided into age-related cortical cataract (ARCC), age-related nuclear cataract (ARNC), and age-related posterior subcapsular cataract (ARPSC) [2]. Cataract surgery, such as phacoemulsification or extracapsular cataract extraction combined with intraocular lens implantation, is regarded as the cost-effective intervention and the only effective approach to treat ARC [2, 3]. However, numbers of annual cataract surgeries vary among countries because of differences in accessibility for diagnosis and surgery, referral, and health-care systems [4, 5]. Several intraoperative complications (e.g., posterior capsule rupture, lens dislocation, and suprachoroidal hemorrhage) and postoperative complications (e.g., refractive error, posterior capsule opacification, endophthalmitis, macular edema, and intraocular pressure elevation) are still exist [6]. Thus, the surgery is unavailable for numerous patients in developing countries, and the surgical complications are not avoided completely [7, 8]. For these reasons, it is compelling to develop novel pharmacological treatment modalities for ARC prevention.

Numerous exogenous and endogenous stimuli, including aging, diabetes, heavy metal exposure, smoking, and UV radiation, give rise to excessive reactive oxygen species (ROS) accumulation, which is a critical risk factor for ARC formation [9–14]. In general, the counteraction of ROS by antioxidants is responsible for redox homeostasis in the transparent lens [15]. However, when the balance is broken due to excessive intracellular ROS level, glutathione (GSH) depletion, and superoxide dismutase (SOD) downregulation, oxidative stress occurs [16]. Increased ROS level results in the apoptosis of lens epithelial cells (LECs), which is regarded as the basis of progression for cataract [17]. Hydrogen peroxide (H_2_O_2_) is one of the most important ROS in damaged LECs. In addition, increased level of H_2_O_2_ is detected in the aqueous humor of ARC patients [18]. Thus, H_2_O_2_ exposure has been widely used to establish the model of ARC ex vivo or in vitro [19, 20].

Nuclear factor kappa B (NF-*κ*B) has been commonly accepted as an important transcription factor, which promotes apoptosis of LECs in response to oxidative damage [21]. In unstimulated cells, NF-*κ*B p65 binds to its inhibitory protein I*κ*B and resides in the cytoplasm in an inactive form. Once I*κ*B is phosphorylated and degraded under exogenous stimuli, NF-*κ*B p65 is translocated into nucleus to regulate the expression of target genes, such as B-cell lymphoma-2 (Bcl-2) gene family and inducible nitric oxide synthase (iNOS) [22, 23]. Bcl-2 family members are divided into proapoptotic molecules (Bax and Bad) and antiapoptotic molecules (Bcl-2 and Bcl-xL), which are responsible for mitochondrial membrane permeability [24]. Meanwhile, iNOS is responsible for generating abundant superoxide anions (O_2_^−^) and nitric oxide (NO). Then, O_2_^−^ reacts with NO for peroxynitrite (ONOO^−^) production, which leads to redox imbalance in LECs [25].

On the other hand, excessive intracellular ROS activates nuclear factor erythroid 2-related factor 2 (Nrf2)/heme oxygenase-1 (HO-1) axis, which is the crucial cellular defense network for redox homeostasis [26]. In case of oxidative stress, Nrf2 was released from Kelch-like ECH-associated protein-1 (Keap-1) and translocated into nucleus to induce the gene expression of HO-1 [27]. HO-1 degrades heme and generates carbon monoxide (CO), ferrous iron (Fe^2+^), and biliverdin (BV) [28]. Previously, our series of studies confirmed that Nrf2/HO-1/CO pathway protects LECs against H_2_O_2_-induced cellular apoptosis [29–31]. Moreover, we established a transgenic HO-1 G143H mouse model of cataract [32]. However, the protection mechanism of Nrf2/HO-1 pathway remains unclear. In addition, our previous study implicated that CO only in part accounts for the cytoprotective property of HO-1 in LECs [29]. Thus, we focused the attention on BV, which is the other product in the catabolic pathway of heme.

Bilirubin (BR), converted by biliverdin reductase A (BVRA) from BV, is regarded as a waste product of the body in the past [33]. However, accumulating evidences suggest that BV and BR redox pair elicits substantial antioxidative and antiapoptotic effects [34]. First, BR is a robust antioxidant [35]. Second, many clinical trials indicated an inverse correlation between circulating total BR concentrations and the risk of several diseases, including diabetic retinopathy, atherosclerosis, cerebrovascular accident, coronary artery disease, and childhood asthma [36–40]. Third, BV/BR redox pair pretreatment shows beneficial effects against oxidative stress and inflammation in large numbers of disease models in vivo and in vitro [41–43]. Furthermore, BR treatment has been reported to activate Nrf2/HO-1 pathway and alleviate nuclear translocation of NF-*κ*B p65 [44–46]. Thus, we hypothesize that BV and BR mediate cytoprotective effect in H_2_O_2_-damaged LECs via regulating NF-*κ*B/iNOS and Nrf2/HO-1 pathway.

In the present study, we compared the relative mRNA expression levels of Nrf2, HO-1, and BVRA in lens capsules of non-cataract and three subtypes of ARC patients. Moreover, the protective role of BV/BR redox pair against oxidative stress was evaluated in cultured mouse LECs.

## 2. Materials and Methods

### 2.1. Human Samples

The anterior lens capsule tissues from ARC patients (*n* = 45 eyes) and age and gender-matched cadaveric human eyes (*n* = 15 eyes) with transparent lens were obtained from the Third Medical Center of PLA General Hospital and the Eye Bank of PLA General Hospital. ARC patients were classified into three subgroups: ARCC (*n* = 15 eyes), ARNC (*n* = 15 eyes), and ARPSC (*n* = 15 eyes). The clinical data of these patients were listed in Table 1.

All participants underwent a detailed ophthalmic examination, including slit lamp microscopy and fundus examination. Cadaveric human eyes were assessed by surgical microscope and indirect ophthalmoscope. The inclusion criteria were as follows: (1) opaque ocular lenses, (2) ≥ 50 years of age, and (3) C ≥ 3, N ≥ 3, or P ≥ 2 according to the lens opacity classification system III (LOCS III). Subjects with a history of intraocular surgery, axial lengths > 26 mm, ocular trauma, glaucoma, uveitis, corneal and retinal pathology, and systemic disorders, such as diabetes mellitus, were excluded from this study.

### 2.2. Cell Culture

The detailed procedures of primary mouse lens epithelial cell culture were consistent with our previous research [32]. In brief, the anterior lens capsule tissues of wild-type C57BL/6 mice (2 months, 20-25g, purchased from Beijing Long'An Animal Center, Beijing, China) were cut into about 1 mm × 1 mm and cultured in Dulbecco's modified Eagle's medium containing 2% fetal bovine serum. The cells were digested and harvested for subculture when they migrate out of the capsule. The cells were sorted and identified by flow cytometry (BD FACSAria, San Jose, CA) with primary antibody against E-cadherin (1 *μ*g/ml, 13-3249-82, Thermo Fisher Scientific, China) and FITC goat polyclonal to biotin (ab16502; Abcam, 1:1000).

### 2.3. Preparation of BV/BR Stock Solution

Biliverdin hydrochloride (30891; Sigma-Aldrich, St. Louis, MO, United States) and bilirubin (B4126; Sigma-Aldrich, St. Louis, MO, United States) were dissolved in 0.2 N NaOH, adjusted to PH of 7.4 with 1 N HCl, and diluted to the final concentration of 10 mM with PBS. The solution was filtered, sterilized, and stored at -80°C.

### 2.4. Cell Transfection

Three small interfering RNA (siRNA) targeting BVRA (siBVRA) and nonsense control (NC) siRNA were synthesized by BoRui Biotech Co., Ltd. (Beijing, China). The sequences of BVRA siRNA are listed in Table S1. Cells were transfected with different siRNA sequences and Lipofectamine 3000™ (Invitrogen).

### 2.5. Cell Counting Kit-8

LECs (5 × 10^3^/well) were seeded in 96-well plates and treated with various concentrations of H_2_O_2_ (50 *μ*M, 100 *μ*M, 200 *μ*M, 400 *μ*M, 800 *μ*M, and 1000 *μ*M), BV, or BR. Then, cells were incubated with 10 *μ*l Cell Counting Kit-8 (CCK-8; Dojindo, Kumamoto, Japan) for 4 h. Cell viability was calculated by examining the absorbance at 450 nm and normalizing to the control group × 100%.

### 2.6. Immunofluorescence Staining

LECs (5 × 10^4^/well) were seeded in 24-well plates. After pretreated with 20 *μ*M BV or BR or 100 *μ*M pyrrolidine dithiocarbamate (PDTC) for 2 h, 200 *μ*M H_2_O_2_ was added to the well for a subsequent 1-h incubation. Then, samples were fixated, incubated with primary antibody against NF-*κ*B p65 (ab16502; Abcam, 1:200) and secondary antibody (ab150077; Abcam, 1:500). PDTC is an effect inhibitor of NF-*κ*B signaling pathway. The reaction was detected using immunofluorescence microscope (Zeiss, German). The fluorescence intensity of nuclear NF-*κ*B p65 was quantified by Image J 1.37v (National Institutes of Health, USA).

### 2.7. Nuclear and Cytoplasmic Extraction

The procedures were determined as reported elsewhere using nuclear extraction kit (ab113474; Abcam, Shanghai, China) [47]. Briefly, cell samples were washed in ice-cold phosphate-buffered saline and centrifuged for 5 min at 1,000 rpm. Then, cells were resuspended in extraction buffer on ice for 10 min and centrifuged for 1 min at 12,000 rpm. After centrifugation, the cytosolic and nuclear fraction were collected and stored at -80°C for western blotting analysis, respectively.

### 2.8. SOD and GSH Detection

Intracellular SOD levels were measured by SOD assay kit-WST (S133; Dojindo, Kumamoto, Japan). The SOD activity was normalized by protein level and calculated according to the reduction rate of WST-1.

Total glutathione quantification kit (T419; Dojindo, Kumamoto, Japan) was used to detect intracellular GSH level by examining the absorbance at 405 nm. The result was calculated by the bicinchoninic acid method with a standard curve.

### 2.9. Flow Cytometry

Flow cytometry was utilized to assess intracellular ROS level, mitochondrial ROS level, and apoptosis. For intracellular ROS level analysis, each cell sample was incubated with 1 ml diluted DCFH-DA (S0033S, Beyotime Biotechnology, Shanghai, China) for 30 min at 37°C. The fluorescence intensity was measured at an excitation wavelength of 488 nm.

The generation of ROS in mitochondria was detected by MitoSOX Red probe (M36008, Thermo Fisher Scientific, China). After treatment, cells were collected and incubated with 5 *μ*M MitoSOX Red probe for 20 min at 37°C. The fluorescence intensity was measured at an excitation wavelength of 510 nm.

For apoptosis analysis, cells (1 × 10^6^/sample) were resuspended, centrifuged, and incubated with 200 *μ*l Annexin V-FITC and 10 *μ*l PI (C1062S, Beyotime Biotechnology, Shanghai, China) for 15 min at room temperature in the dark. Then, the apoptotic and dead cells were calculated using flow cytometry.

### 2.10. Quantitative Real-Time Polymerase Chain Reaction (qPCR)

The lens capsule samples were homogenized using ultrasonic homogenizer. TRIzol™ reagent (15596026; Thermo Fisher Scientific, Shanghai, China) was used for RNA extraction. After evaluating RNA concentration and purity, PCR reactions were conducted using PowerTrack™ SYBR Green Master Mix (A46012; Thermo Fisher Scientific, Shanghai, China) by StepOne™ Real-Time PCR system (Thermo Fisher Scientific, Shanghai, China). The PCR program was 10 min at 95°C, 40 cycles of 94°C for 15 min, and 60 s at 60°C. Relative gene expression levels were determined by the 2 ^(-*ΔΔ*Ct)^ method. GAPDH was used an internal standard, and the gene expression was normalized to this. All primer sequences used in this study were listed in Table S2.

### 2.11. Western Blotting Analysis

LECs were lysed, and protein concentration was evaluated using BCA protein assay kit (P0012, Beyotime Biotechnology, Shanghai, China). Then, protein samples were added to SDS-PAGE and blotted onto PVDF membrane. Primary antibodies against NF-*κ*B p65 (ab16502; Abcam, 1:1000), I*κ*B (ab32518; Abcam, 1:1000), iNOS (ab178945; Abcam, 1:1000), Bax (ab32503; Abcam, 1:1000), Bcl-2 (ab182858; Abcam, 1:1000), Caspase-3 (ab184787; Abcam, 1:1000), BVRA (sc-393385; Santa Cruz, 1:1000), Nrf2 (ab92946; Abcam, 1:1000), HO-1 (ab52947; Abcam, 1:1000), GAPDH (ab8245; Abcam, 1:5000), and histone H3 (ab1791; Abcam, 1:5000) were incubated with blots at 4°C overnight. Then, blots were incubated with goat anti-mouse IgG H&L (ab150113; Abcam, 1:5000) or goat anti-rabbit IgG H&L (ab150077; Abcam, 1:5000) for 1 h at room temperature. The blots were detected by BeyoECL Plus kit (P0018S, Beyotime Biotechnology, Shanghai, China) and analyzed using Image J software. GAPDH was used as a cytosolic marker and H3 a nuclear marker.

### 2.12. Statistical Analysis

The sample size of enrolled participants in this study was determined using PASS 15 software (NCSS, LLC. Kaysville, Utah, USA). Data were expressed as the mean ± standard error of mean (SEM) and analyzed by GraphPad Prism 9.2 software (San Diego, CA, USA). Each experiment was duplicated for triple times. All results were analyzed with chi-square test or one-way ANOVA with post hoc least significant difference test. *P* < 0.05 was considered for statistically significant difference.

## 3. Results

### 3.1. Decreased Expression of Nrf2, HO-1, and BVRA in the Lens Capsule of ARNC Patients

**Figure 1:**
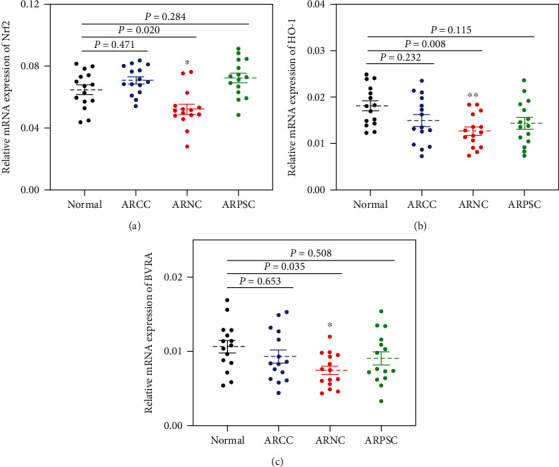
Decreased levels of Nrf2, HO-1 and BVRA in lens capsule were associated to age-related nuclear cataract. The relative mRNA expression levels of Nrf2 (a), HO-1 (b), and BVRA (c) in anterior lens capsule tissues of normal subjects, age-related cortical cataract, age-related nuclear cataract, and age-related posterior subcapsular cataract were measured using qPCR. Data are shown as mean ± SEM, *n* = 15 in each group, one-way ANOVA, ∗*P* < 0.05, ∗∗*P* < 0.01, compared with the normal group.

To determine the role of Nrf-2, HO-1, and BVRA in three subtypes of ARCs, we assessed the expression levels of Nrf2, HO-1, and BVRA in the anterior lens capsule of human samples. Compared with non-cataract patients, the relative mRNA expressions of Nrf2, HO-1, and BVRA were all decreased in the anterior lens capsule of the age and gender-matched ARNC (*P* = 0.020, *P* = 0.008, *P* = 0.035, Figures 1(a)–1(c)). However, the data showed no difference in LECs among the normal subjects, ARCC and ARPSC (all *P* > 0.05).

### 3.2. BV and BR Counteract H_2_O_2_-Induced Cytotoxicity in LECs

The cytotoxic effect of H_2_O_2_, BV, and BR in mouse LECs was determined by CCK-8 assay. The cells were incubated with various concentrations of H_2_O_2_ (50 *μ*M to 1000 *μ*M) or BV or BR (5 *μ*M to 500 *μ*M) for 24 h. As shown in Figure S1(a), H_2_O_2_ had a dose-dependent inhibition of cell viability, and the half-maximal inhibitory concentration was 200 *μ*M. When the concentration of BV or BR was over 100 *μ*M, cell viability was dramatically decreased compared with the control group (Figure S1(b)).

To determine the cytoprotective property of BV or BR against H_2_O_2_-induced cell damage, BV/BR (5 *μ*M to 100 *μ*M) were pretreated for 2 h before LECs being exposed to 200 *μ*M H_2_O_2_ for 24 h. Compared with H_2_O_2_-treated group, BV or BR pretreatment (5 *μ*M to 50 *μ*M) recovered the cell viability damaged by H_2_O_2_ significantly. Among them, cells pretreated with 20 *μ*M BV or BR showed the highest viability (Figure S1(c)). Based on these results, 200 *μ*M H_2_O_2_ and 20 *μ*M BV or BR were chosen for subsequent experiments.

### 3.3. BV/BR Redox Pair Prohibits the Activation of NF-*κ*B/iNOS Signaling Pathway

**Figure 2:**
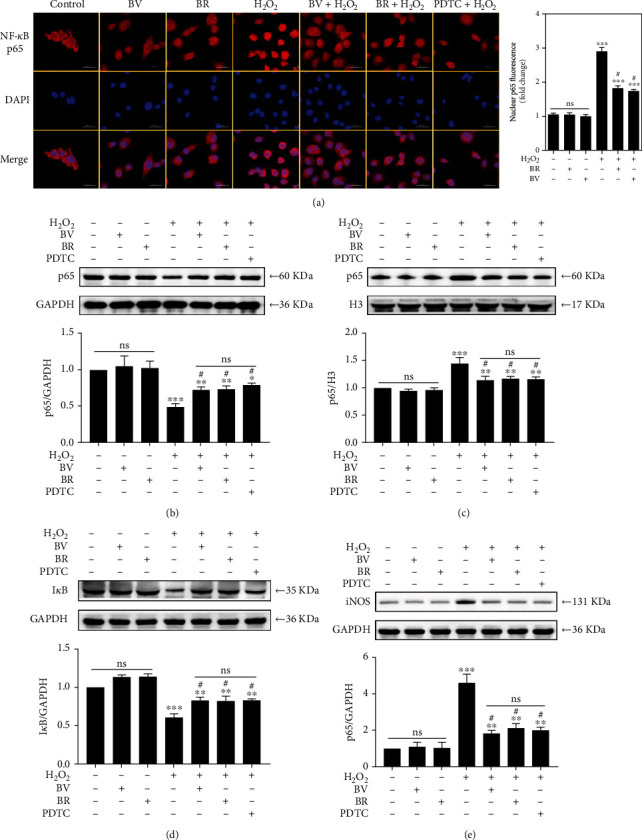
BV/BR pretreatment prohibited H_2_O_2_-induced activation of NF-*κ*B/iNOS pathway. LECs were pretreated with 20 *μ*M BV/BR or 100 *μ*M PDTC for 2 h before being exposed to 200 *μ*M H_2_O_2_ for 1 h. (a) Cells were stained with DAPI (blue) and immunostained with NF-*κ*B p65 antibody (red). The relative expressions of NF-*κ*B p65 in cytosolic (b) and nuclear (c) fractions, I*κ*B (d), and iNOS (e) were determined by western blotting analysis. Data are shown as mean ± SEM, *n* = 3, one-way ANOVA, ∗*P* < 0.05, ∗∗*P* < 0.01, ∗∗∗*P* < 0.001, compared with the control group; #*P* < 0.05, compared with the H_2_O_2_-treated group. Bar 50 *μ*m.

The activation of NF-*κ*B/iNOS signaling pathway was detected by immunofluorescence staining and western blotting analysis. When cells were incubated with 20 *μ*M BV or BR alone for 2 h, NF-*κ*B p65 (red channel) was mainly retained in cytoplasm. When cells were exposed to 200 *μ*M H_2_O_2_ for 1 h, the cytosolic NF-*κ*B p65 translocated into the nucleus (blue channel), with a 3-fold higher fluorescence intensity of nuclear NF-*κ*B p65 compared with the control group (*P* < 0.001). However, cells pretreated with 20 *μ*M BV or BR or 100 *μ*M PDTC for 2 h decreased the fluorescence intensity of nuclear NF-*κ*B p65 to about 1.8 and 1.6-fold than those of the H_2_O_2_-treated group, respectively (*P* < 0.05). Thus, BV, BR, and PDTC pretreatment prohibited the H_2_O_2_-triggered NF-*κ*B p65 nuclear trafficking (Figure 2(a)).

Compared with the control group, NF-*κ*B p65 nuclear trafficking and iNOS expression were not remarkably affected in both BV and BR groups by western blotting analysis. Cells incubated with 200 *μ*M H_2_O_2_ for 1 h leads to an increase of nuclear NF-*κ*B p65 and total iNOS expression and a decrease of cytosolic NF-*κ*B p65 and total I*κ*B expression (*P* < 0.001). Nevertheless, the effect was attenuated by the pretreatment with 20 *μ*M BV or BR or 100 *μ*M PDTC for 2 h (*P* < 0.05, Figures 2(b)–2(e)).

### 3.4. BV and BR Improve the Intracellular Redox Homeostasis

**Figure 3:**
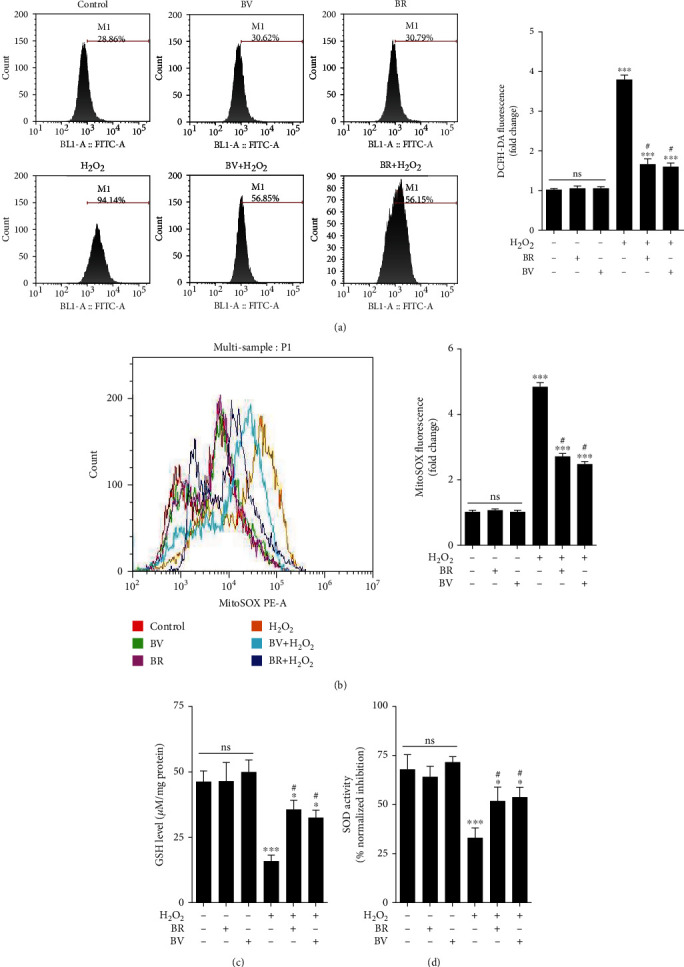
BV/BR pretreatment decreased intracellular ROS levels and restores antioxidants levels. (a) Intracellular ROS levels in each cell sample were detected using a cell permeable probe, DCFH-DA. (b) Mitochondrial ROS levels in each cell sample were detected by MitoSOX Red probe. (c) GSH levels were detected by total glutathione quantification kit. (d) SOD activity was measured by SOD assay kit-WST. Data are shown as mean ± SEM, *n* = 3, one-way ANOVA, ∗*P* < 0.05, ∗∗∗*P* < 0.001, compared with the control group; #*P* < 0.05, compared with the H_2_O_2_-treated group.

Intracellular ROS levels were detected by DCFH-DA staining. It was obvious that ROS production is approximately 4-fold higher in cells damaged by 200 *μ*M H_2_O_2_ for 24 h than that in the control group (*P* < 0.001). Correspondingly, pretreatment of LECs with BV or BR at 20 *μ*M for 2 h significantly decreased the ROS level to about 1.7 and 1.8-fold compared to that in H_2_O_2_-treated group, respectively (*P* < 0.05, Figure 3(a)).

In addition, mitochondrial ROS levels were measured with MitoSOX Red probe. The ROS level in mitochondria was about 5-fold higher in cells treated with 200 *μ*M H_2_O_2_ compared with that in the control group (*P* < 0.001). However, 20 *μ*M BV and BR pretreatment decreased the mitochondrial ROS levels to about 2.8 and 2.5-fold than those in H_2_O_2_-treated group, respectively (*P* < 0.05, Figure 3(b)). Compared to the control group, H_2_O_2_ treatment significantly decreased the GSH level and SOD activity (both *P* < 0.001). However, BV or BR pretreatment at 20 *μ*M for 2 h restored the GSH level and SOD activity compared to the H_2_O_2_-exposed group, respectively (both *P* < 0.05, Figures 3(c) and 3(d)). BV or BR alone treatment at 20 *μ*M had no effect on intracellular ROS, GSH levels and SOD activity in LECs.

### 3.5. BV/BR Redox Pair Attenuates the Apoptotic Rate of H_2_O_2_-Damaged LECs

**Figure 4:**
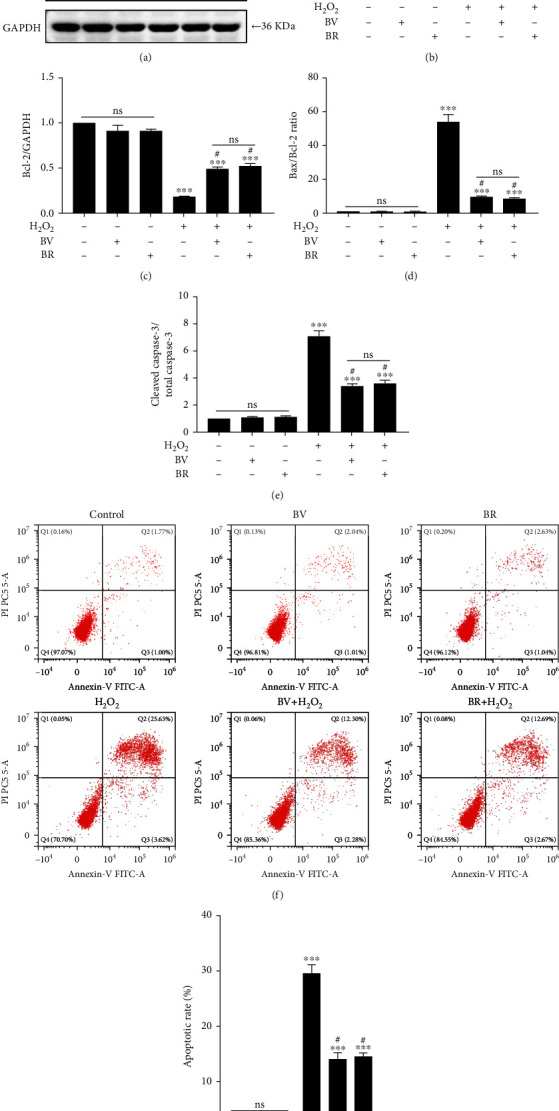
BV/BR pretreatment suppressed the apoptotic rate in H_2_O_2_-damaged LECs. (a) The representative bands of the apoptotic molecules (Bax, Bcl-2, and cleaved caspase-3) were determined with western blotting analysis. (b–e) The apoptotic molecules were semi-quantified by densitometry and analyzed with GAPDH as reference. (f) The representative images of the apoptotic rates in LECs by Annexin V-FITC assay. (g) The statistic results of cellular apoptotic rates. Data are shown as mean ± SEM, *n* = 3, one-way ANOVA, ∗∗∗*P* < 0.001, compared with the control group; #*P* < 0.05, compared with the H_2_O_2_-treated group.

The relative expressions of the apoptotic proteins (Bax, Bcl-2, and cleaved caspase-3) were determined using western blotting analysis. Compared to the control group, after H_2_O_2_ exposure at 200 *μ*M for 24 h, the Bcl-2 level decreased to 0.12-fold (*P* < 0.001), while the levels of Bax and cleaved caspase-3 increased to about 10 and 6-fold (both *P* < 0.001), respectively. Compared to the H_2_O_2_ alone group, BV or BR pretreatment at 20 *μ*M for 2 h restored Bcl-2 level to about 0.5-fold and decreased the Bax and cleaved caspase-3 levels to approximately 5 and 4-fold, respectively (all *P* < 0.05, Figures 4(a)–4(e)). There was no obvious change of Bax, Bcl-2, and cleaved caspase-3 levels in the BV or BR alone treatment group, compared to that of the control group.

The protective effect of BV and BR against H_2_O_2_-induced cell apoptosis were determined by Annexin V-FITC assay. The H_2_O_2_-treated group showed a higher apoptotic rate (29.57 ± 0.92) compared with the control group (2.80 ± 0.36, *P* < 0.001). However, BV or BR pretreatment at 20 *μ*M decreased the apoptotic rate to 14.55 ± 0.34 and 14.10 ± 0.64 compared to the H_2_O_2_ alone group, respectively (both *P* < 0.05, Figures 4(f) and 4(g)). BV or BR alone treatment at 20 *μ*M had no effect on cellular apoptosis.

### 3.6. Cytoprotective Effect of BV Was Substantially Suppressed by Knockdown of BVRA

**Figure 5:**
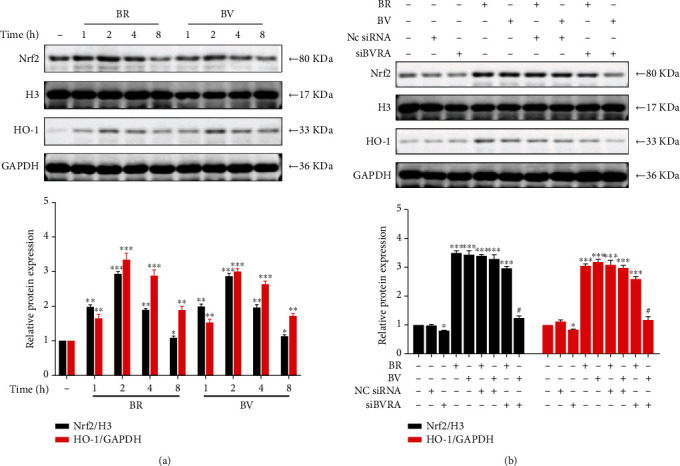
BV-mediated activation of Nrf2/HO-1 pathway is diminished by knocking down BVRA. (a) LECs were treated with BV (20 *μ*M) or BR (20 *μ*M) for various time. The representative bands of HO-1 and nuclear translocated Nrf2 were shown by western blotting analysis and semi-quantified with GAPDH or histone H3 as reference. (c) After being transfected with BVRA siRNA or NC siRNA, LECs were treated with BV (20 *μ*M) or BR (20 *μ*M) for 2 h. The representative bands of HO-1 and nuclear translocated Nrf2 were shown by western blotting analysis and semi-quantified with GAPDH or histone H3 as reference. Data are shown as mean ± SEM, *n* = 3, one-way ANOVA, ∗*P* < 0.05, ∗∗*P* < 0.01, ∗∗∗*P* < 0.001, compared with the control group; #*P* < 0.01, compared with the BR-treated group.

**Figure 6:**
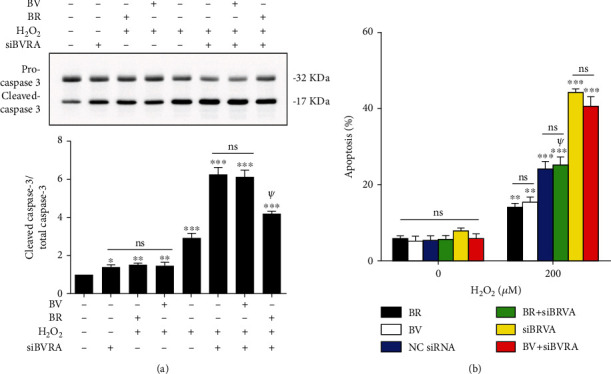
The antiapoptotic property of BV in LECs was substantially suppressed by knockdown of BVRA. (a) After silencing BVRA expression, LECs were pretreated with BV/BR (20 *μ*M) for 2 h or damaged with H_2_O_2_ (200 *μ*M) for 24 h. The representative bands of cleaved caspase-3 were shown by western blotting analysis and semi-quantified with GAPDH as reference. (b) The apoptotic rates of LECs in various groups were measured by Annexin V-FITC assay. Data are shown as mean ± SEM, *n* = 3, one-way ANOVA, ∗*P* < 0.05, ∗∗*P* < 0.01, ∗∗∗*P* < 0.001, compared with the control group; *ψP* < 0.01, compared with the siBVRA+H_2_O_2_ group.

To further determine the protective effect of BV/BR redox pair against oxidative stress in LECs, gene expression of BVRA was silenced by siRNA transfection. As a result, 2# siBVRA transfection led to an approximately 70% decrease of BVRA mRNA expression compared to the control cells (Figure S2), and it was selected for subsequent experiments. Both BV and BR treatment at 20 *μ*M suggested that the relative protein expression of nuclear Nrf2 and total HO-1 reached the peak at 2 h in LECs (Figure 5(a)). As shown in Figure 5(b), nuclear accumulation of Nrf2 and total HO-1 expression by 20 *μ*M BV treatment were prohibited in siBVRA transfected cells. In addition, knockdown of BVRA canceled out the effect of BV on decreasing the expression of cleaved caspase-3 (Figure 6(a)) and the apoptotic rate (Figure 6(b)) in H_2_O_2_-treated LECs. Nevertheless, BVRA silencing had no cellular effect in BR-pretreated cells. All the data elucidated that BVRA is responsible for the cytoprotective effect of BV against oxidative damage in LECs by reductive conversion of BV to BR.

## 4. Discussion

**Figure 7:**
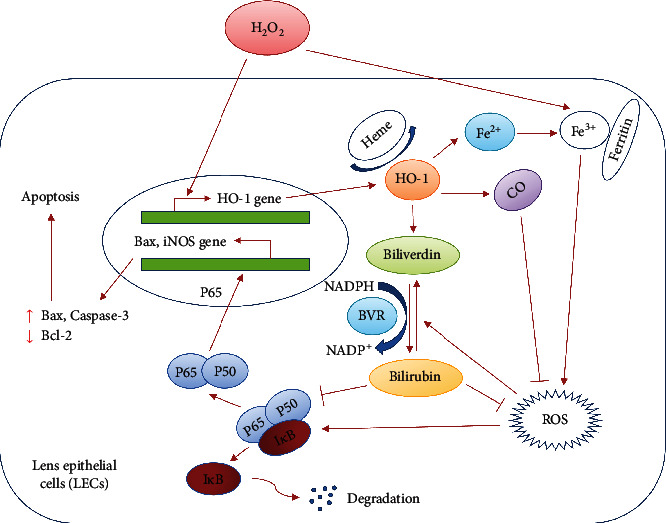
The crosstalk between HO-1 and oxidative stress in LECs. The scheme reveals that HO-1 degrades heme and generates biliverdin, carbon monoxide, and ferrous iron (Fe^2+^). Biliverdin is converted to bilirubin by biliverdin reductase. Both carbon monoxide and biliverdin/bilirubin redox pair act as ROS scavengers by suppressing the activation of NF-*κ*B/iNOS pathway. Ferrous iron is a pro-oxidant, which enhances ROS generation by Fenton reaction. Nevertheless, the elevated levels of ferrous iron can also lead to the upregulation expression of ferritin, which exerts the antioxidative property.

In the present study, we found that the relative gene expressions of Nrf2, HO-1, and BVRA are decreased in the anterior capsule of ARNC patients. Moreover, BV/BR redox pair improves the intracellular redox homeostasis and protects against H_2_O_2_-induced apoptosis in lens epithelial cells. The cytoprotective effect was mediated by downregulating NF-*κ*B/iNOS pathway and activating Nrf2/HO-1 pathway. Together with our previous work, the present study confirmed that Nrf2/HO-1/BV/BVRA/BR axis plays an important role in attenuating oxidative stress both in LECs of ARC patients and in H_2_O_2_-exposure conditions (Figure 7).

The pathogenesis of three subtypes of ARC is not same [48]. For example, oxidative stress, especially GSH depletion, is regarded as the hallmark of ARNC [16]. Ionic imbalance caused by loss of Na^+^/K^+^-ATPase function and water content increase is the proposed mechanism for cortical cataract genesis, while ARPSC is more common in patients with retinitis pigmentosa, diabetes, myopia, and long-term use of corticosteroids [49, 50]. Thus, it is no surprise that the relative mRNA expressions of Nrf2, HO-1, and BVRA, which are crucial for resisting oxidative stress, were all decreased in the LECs of ARNC patients.

Since excessive accumulation of BR during neonatal life gives rise to serious neurological damage, BR has long been considered as a cytotoxic substance [51]. Nevertheless, substantial studies currently suggest a cytoprotective effect for BV/BR redox pair [35, 38, 39, 42] In our present study, the data indicated that 20 *μ*M BV or BR pretreatment for 2 h has a potent protective effect in H_2_O_2_-damaged mouse LECs. In addition, the cytotoxicity of BV or BR was significant over the concentration of 100 *μ*M. However, whether this concentration coincides with the endogenous levels of BV and BR in lens tissues needs further evaluation.

Stable intracellular redox status is a key factor to prevent cataract formation [52, 53]. In contrast to the aerobic respiratory tissues, ROS in the lens tissue is mainly produced from endoplasmic reticulum stress-triggered chronic unfolded protein response [54]. In general, most of the endogenous and exogenous stressors are mild and give rise to physiological levels of ROS, which is crucial for cell protection throughout the lifespan [55]. However, once the cells were exposed to weak levels of stressors chronically or severe stressors in a short time, it leads to a shift in the balance between oxidative damage and antioxidant defenses [56]. Excessive intracellular ROS accumulation and overwhelmed antioxidant capacity induce misfolded protein aggregation in the lens and LECs apoptosis, which is one of the reasons for ARC formation [57, 58]. In the present study, we found that 200 *μ*M H_2_O_2_ exposure in mouse LECs for 24 h results in an increase of intracellular ROS level and depletion of GSH and SOD. However, these changes were attenuated by preconditioning of 20 *μ*M BV or BR for 2h, which suggests that BV/BR redox pair has a function of restoring intracellular redox homeostasis.

The transcription factor NF-*κ*B plays a profound role in regulating the expression of genes involved in the response of inflammation and oxidative stress [27, 59]. Enhanced cytosolic H_2_O_2_ levels result in NF-*κ*B signaling pathway activation by promoting NF-*κ*B p65 nuclear translocation and I*κ*B degradation [60, 61]. As shown in our data (Figure 2), NF-*κ*B p65 is mainly retained in the cytoplasm of untreated LECs. After H_2_O_2_ exposure, NF-*κ*B p65 was obviously translocated to nuclei with a decreased expression of total I*κ*B, which is in accordance with the data from another study [21]. However, BV/BR redox pair and PDTC pretreatment dramatically prohibited the NF-*κ*B p65 nuclear accumulation and I*κ*B degradation induced by H_2_O_2_ damage.

Both iNOS and Bcl-2 gene family (Bax, Bcl-2, Bcl-xL, and Bad) are the key downstream target genes of NF-*κ*B pathway [62, 63]. Jia et al. indicated that NF-*κ*B p65 accumulated in nuclei binds to iNOS promoter by chromatin immunoprecipitation assay [25]. Under oxidative condition, intracellular iNOS is induced and products abundant in superoxide anions (O_2_^−^) and nitric oxide (NO). Then, O_2_^−^ reacts with NO for ONOO^−^ production [64]. The oxidative capacity of ONOO^−^ is over 2000-fold stronger than H_2_O_2_, which brings about the exacerbation of redox homeostasis in LECs [65]. In the present study, our results verified that NF-*κ*B p65 nuclear translocation induced by H_2_O_2_ exposure leads to the increased expression of iNOS. Nevertheless, BV or BR pretreatment ameliorated the expression of iNOS.

The intracellular ROS levels also regulate the expression of Bcl-2 family members, which is responsible for mitochondrial membrane permeability [66]. Bcl-2 family members are divided into proapoptotic molecules (Bax and Bad) and antiapoptotic molecules (Bcl-2 and Bcl-xL) [67]. As shown in our previous work, excessive ROS leads to mitochondrial-mediated apoptosis in LECs, which is characterized by dissipation of mitochondrial membrane potential, mitochondrial membrane rupture, upregulation of Bax and cleaved caspase-3, and downregulation of Bcl-2 [20]. In the present study, we found that BV or BR pretreatment can partially restores the protein expression of Bcl-2, Bax, and cleaved caspase-3.

In general, the reductive conversion of BV to BR by BVRA is an energy-consuming and evolutionarily conserved process in human physiology [68]. Therefore, we assumed that BVRA is essential for BV to carry out cellular function. To verify this hypothesis, gene expression of BVRA was silenced by siRNA transfection. As shown in Figure 5 and Figure 6, the data revealed that BR markedly promotes HO-1 induction and Nrf2 nuclear accumulation, whereas the activation of Nrf2/HO-1 pathway by BV is dependent on BVRA. In addition, BVRA silencing counteracted the antiapoptotic effect of BV on H_2_O_2_-treated LECs. Nevertheless, BVRA knockdown had no cellular effect in BR-pretreated cells. Taken all these into consideration, BVRA is essential for cytoprotective effect of BV by converting BV to BR. Moreover, the rapid reductive conversion of BV to BR by BVRA in higher organism, which is most subject to exogenous and endogenous stressors such as excessive oxidation, is thought to be a metabolic evolution for sophisticated physiology [69].

There were several limitations in the present study. (1) Due to the limited cell numbers in human anterior lens capsule, we cannot measure the concentrations of BV and BR by commercial colorimetric assay kit directly. As an alternative, we have to determine the expression levels of enzymes regulating BV and BR generation, such as Nrf2, HO-1, and BVRA. The data cannot explain whether there are BV and BR in human lens and aqueous humor. (2) There were only data from human samples and mouse LECs in the present study. In the future, the protective effect of BV and BR against cellular senescence should be investigated in a naturally aged mouse model in vivo.

## 5. Conclusions

To sum up, BV/BR redox pair protects LECs against oxidative stress-induced apoptosis by promoting intracellular redox homeostasis, suppressing NF-*κ*B/iNOS pathway, and activating Nrf2/HO-1 pathway. Moreover, BVRA is responsible for the cytoprotective effect of BV against oxidative damage by reductive conversion of BV to BR.

## Figures and Tables

**Table 1:** Demographic of patients with or without age-related cataract (ARC).

	Normal (*n* = 15)	ARCC (*n* = 15)	ARNC (*n* = 15)	ARPSC (*n* = 15)	*P* value
Age (years)	60.00 ± 1.68	58.20 ± 1.69	58.87 ± 1.37	62.2 ± 1.28	0.272^a^
Gender (male/female)	9/6	7/8	8/7	5/10	0.506^b^

ARCC: age-related cortical cataract; ARNC: age-related nuclear cataract; ARPSC: age-related posterior subcapsular cataract. ^a^: one-way ANOVA; ^b^: chi-square test.

## Data Availability

All data generated and analyzed in the present study are available from the corresponding author on reasonable request.

## Supplementary Materials

Supplementary 1 The sequences of siRNA are shown in Table S1. The primer pairs for qPCR are listed in Table S2.

Supplementary 2 Supplementary Figure S1: Effect of H_2_O_2_, BV, and BR on mouse LECs. (a) Cell viability of LECs treated with various concentrations of H_2_O_2_ for 24 h was tested using CCK-8 assay. (b) Cytotoxicity of different concentrations of BV/BR for 24 h was tested using CCK-8 assay. (c) Cell viability of LECs pretreated with different concentrations of BV/BR (2 h) under 200 *μ*M H_2_O_2_ treatment (24 h) was determined by CCK-8 assay. Data are shown as mean ± SEM, *n* = 3, one-way ANOVA, ∗*P* < 0.05, ∗∗*P* < 0.01, ∗∗∗*P* < 0.001, compared with the control group.

Supplementary 3 Supplementary Figure S2: Gene silence effect of different siRNA sequences (NC siRNA, 1#, 2#, and 3#) on relative mRNA expression of BVRA in LECs was determined by qPCR. Data are shown as mean ± SEM, *n* = 3, one-way ANOVA, ∗*P* < 0.05, ∗∗∗*P* < 0.001, compared with the control group.
