# Supplementary material for: Biliverdin/Bilirubin Redox Pair Protects Lens Epithelial Cells against Oxidative Stress in Age-Related Cataract by Regulating NF-κB/iNOS and Nrf2/HO-1 Pathways
Source: Oxid Med Cell Longev. 2022 Apr 15;2022:7299182. doi: 10.1155/2022/7299182 (PMC9036166; doi:10.1155/2022/7299182)
Supplement: Supplementary 1 — The sequences of siRNA are shown in Table S1. The primer pairs for qPCR are listed in Table S2. [file 7299182.f1.docx]

**Supplementary Materials**

**Table S1:** **The sequences of BVRA siRNA and NC siRNA**

|  | Sense (5’→3’) | Antisense (5’→3’) |
| --- | --- | --- |
| siBVRA 1# | GCCUAUAUUUGCACUGAGAGU | UCUCAGUGCAAAUAUAGGCGA |
| siBVRA 2# | CUUACAGUGUUGACAUCUAAA | UAGAUGUCAACACUGUAAGGG |
| siBVRA 3# | GCUUCAGAUACCUCUGAAACG | UUUCAGAGGUAUCUGAAGCUU |
| NC siRNA | UUCUCCGAACGUGUCACGUTT | ACGUGACACGUUCGGAGAATT |

**Table S2: The sequences of primer pairs for qPCR**

| Gene | Forward (5’→3’) | Reverse (5’→3’) |
| --- | --- | --- |
| HO-1 (human) | GGTCCTTACACTCAGCTTTCT | CATAGGCTCCTTCCTCCTTTC |
| BVRA (human) | TAATGCTGGCAAGCACGT | GGTCTTTCCCCACCACTTCT |
| Nrf2 (human) | TCAGCGACGGAAAGAGTATGA | CCACTGGTTTCTGACTGGATGT |
| GAPDH (human) | GGAGCGAGATCCCTCCAAAAT | GGCTGTTGTCATACTTCTCATGG |
| BVRA (mouse) | GAAAGGGAGAGTCCTGCATGA | CTGGCTGTGAAGCGAAGAGAT |
| GAPDH (mouse) | TGGATTTGGACGCATTGGTC | TTTGCACTGGTACGTGTTGAT |


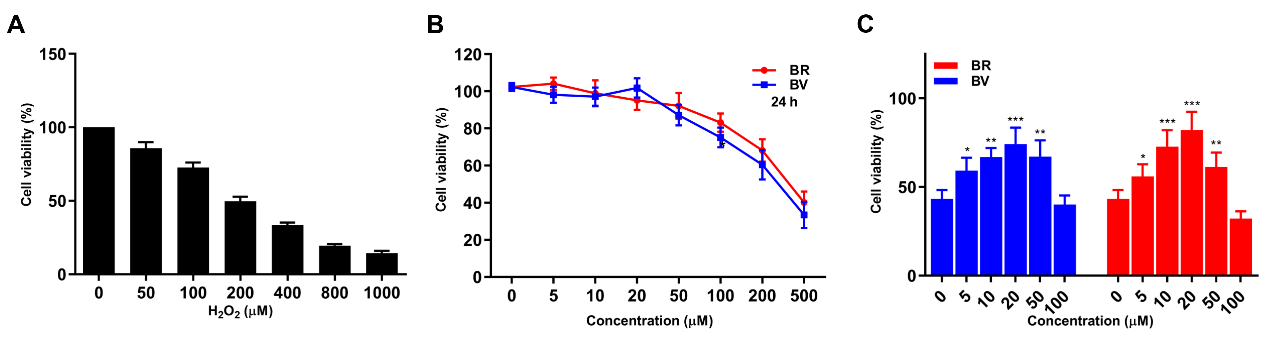


**FIGURE S1:** Effect of H_2_O_2_, BV, and BR on mouse LECs. (a) Cell viability of LECs treated with various concentrations of H_2_O_2_ for 24 h was tested using CCK-8 assay. (b) Cytotoxicity of different concentrations of BV/BR for 24 h was tested using CCK-8 assay. (c) Cell viability of LECs pretreated with different concentrations of BV/BR (2 h) under 200 μM H_2_O_2_ treatment (24 h) was determined by CCK-8 assay. Data are shown as mean ± SEM, n=3, one-way ANOVA, *P<0.05, **P<0.01, ***P<0.001, compared with the control group.


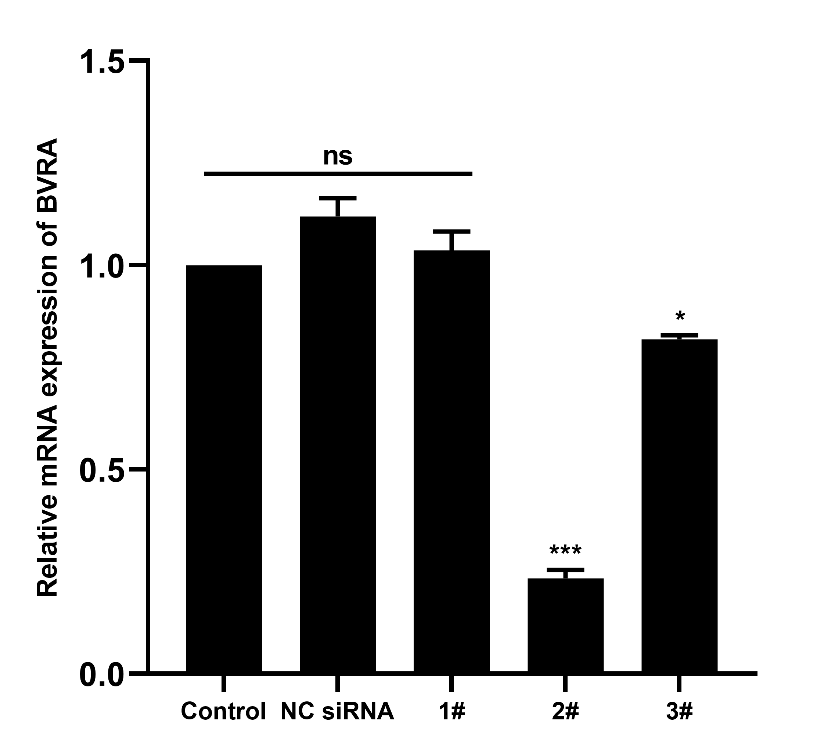


**FIGURE S2:** Gene silence effect of different siRNA sequences (NC siRNA, 1#, 2#, and 3#) on relative mRNA expression of BVRA in LECs was determined by qPCR.
