# Supplementary figures and images for: Biliverdin/Bilirubin Redox Pair Protects Lens Epithelial Cells against Oxidative Stress in Age-Related Cataract by Regulating NF-κB/iNOS and Nrf2/HO-1 Pathways
Source: Oxid Med Cell Longev. 2022 Apr 15;2022:7299182. doi: 10.1155/2022/7299182 (PMC9036166; doi:10.1155/2022/7299182)

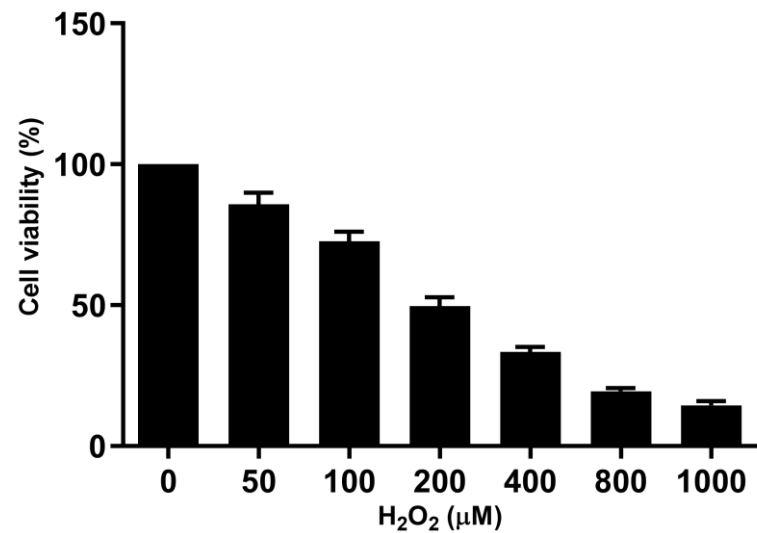

(a)

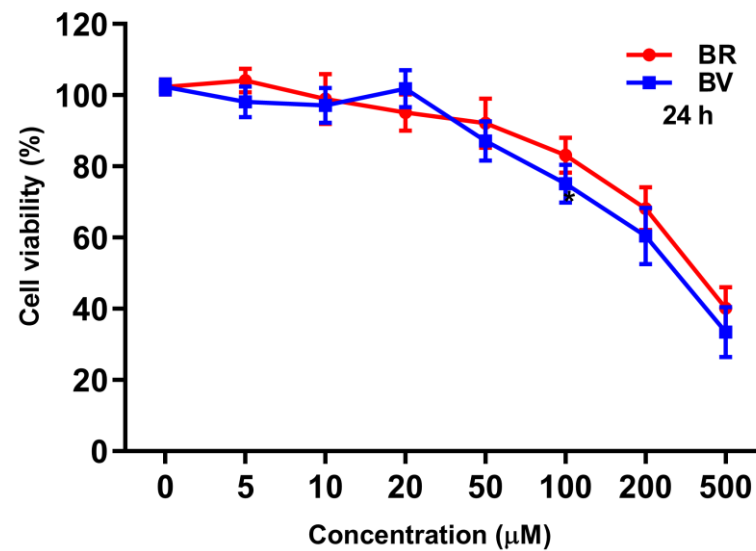

(b)

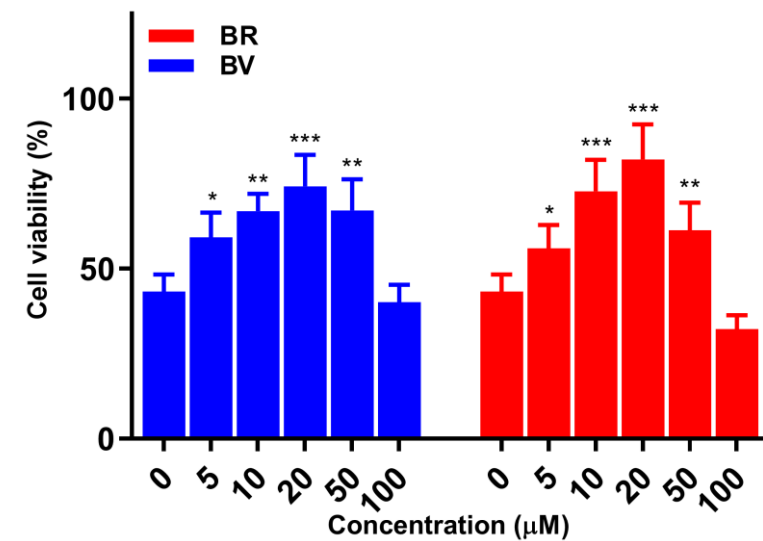

(c)

Supplement: Supplementary 2 — Supplementary Figure S1: Effect of H2O2, BV, and BR on mouse LECs. (a) Cell viability of LECs treated with various concentrations of H2O2 for 24 h was tested using CCK-8 assay. (b) Cytotoxicity of different concentrations of BV/BR for 24 h was tested using CCK-8 assay. (c) Cell viability of LECs pretreated with different concentrations of BV/BR (2 h) under 200 μM H2O2 treatment (24 h) was determined by CCK-8 assay. Data are shown as mean ± SEM, n = 3, one-way ANOVA, ∗P < 0.05, ∗∗P < 0.01, ∗∗∗P < 0.001, compared with the control group. [file 7299182.f2.pdf]

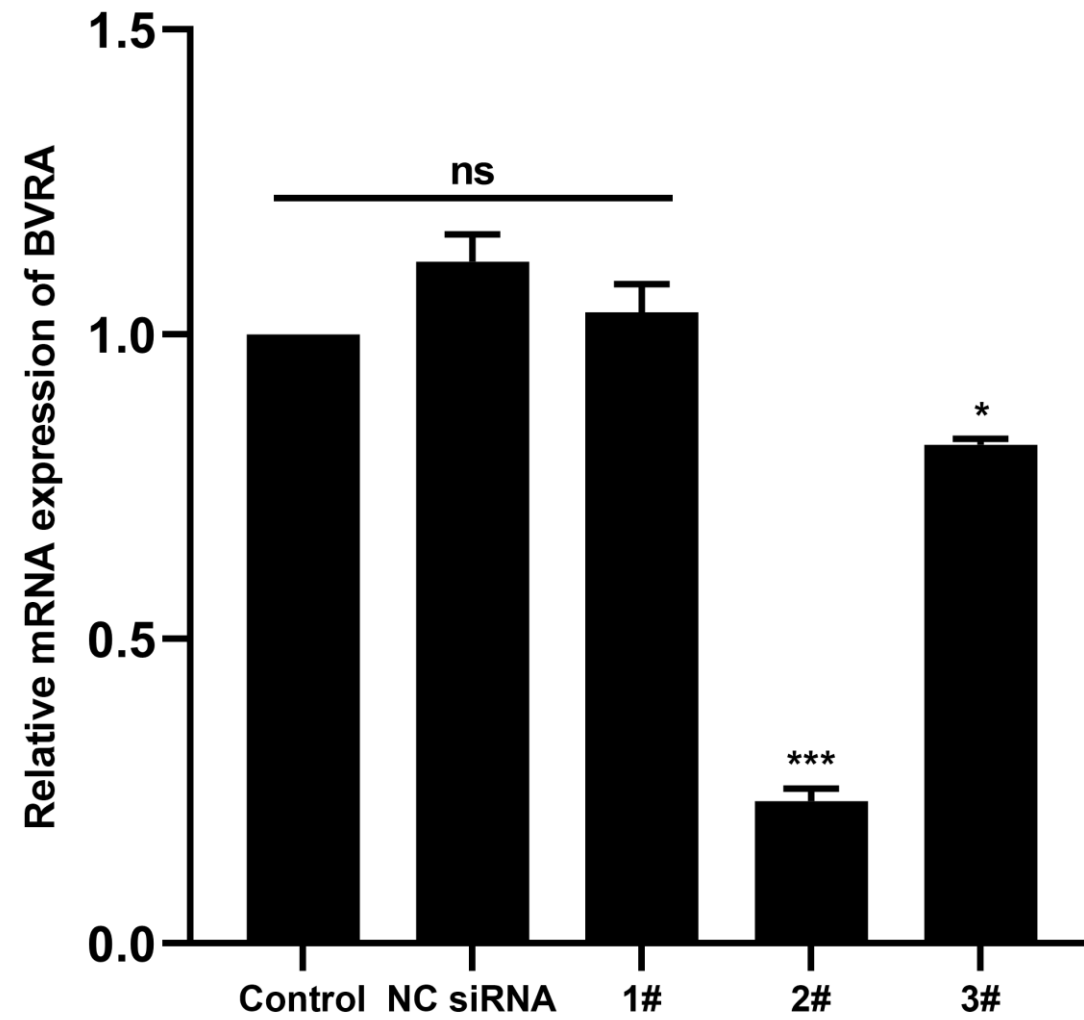

Supplement: Supplementary 3 — Supplementary Figure S2: Gene silence effect of different siRNA sequences (NC siRNA, 1#, 2#, and 3#) on relative mRNA expression of BVRA in LECs was determined by qPCR. Data are shown as mean ± SEM, n = 3, one-way ANOVA, ∗P < 0.05, ∗∗∗P < 0.001, compared with the control group. [file 7299182.f3.pdf]
